# Adolescent dating violence measurement and psychometric terminology: a bibliometric review

**DOI:** 10.3389/fpsyg.2026.1845427

**Published:** 2026-09-24

**Authors:** Andrés Ramírez

**Affiliations:** Carrera de Psicología Clínica, Grupo de Investigación en Neurociencia Clínica Aplicada (GINCA), Universidad Politécnica Salesiana, Cuenca, Ecuador

**Keywords:** adolescent, bibliometric review, dating violence, measurement instruments, psychometric scales

## Abstract

Adolescent dating violence is a multidimensional public health problem encompassing physical, sexual, psychological, relational, coercive, stalking, and technology-facilitated behaviors within current or former dating relationships. As prevalence estimates, risk profiles, prevention priorities, and clinical decisions depend on how the construct is defined and operationalized, the organization of the measurement literature warrants systematic mapping. This bibliometric review mapped scientific production at the intersection of adolescent dating violence and measurement or psychometric terminology. A search was conducted on the Web of Science and Scopus on 20 March 2026, and a final corpus of 6,125 bibliographic records published from 1987 to 2026 was analyzed using *bibliometrix* package in *R*. The analysis focused on temporal growth, sources, authors, institutions, countries, keyword co-occurrence, and conceptual structure; a logistic model was used to summarize the cumulative production of literature. The output increased markedly after 2014 and reached 802 records in 2025. The logistic model yielded a projected upper asymptote of *K =* 7,861.20 records and a midpoint of *tm =* 36.44 years from the 1986 baseline (approximately 2022.44), with *R^2^ =* 0.87. These estimates were interpreted cautiously because of residual deviations and incomplete 2026 coverage. The literature was concentrated in journals addressing interpersonal violence, psychology, mental health, and assessment. Thematic mapping connected validation, reliability, adolescent development, mental health, gender, aggression, and prevalence. However, cross-cultural adaptation beyond highly represented regions and inclusive measurement for lesbian, gay, bisexual, transgender, queer or questioning, intersex, asexual, non-binary, and other sexual and gender identities (LGBTQIA+) adolescents remained peripheral. The field is expanding and moving toward consolidation, but publication volume should not be equated with methodological maturity.

## Introduction

Adolescent dating violence refers to abusive or controlling behaviors that occur within current or former romantic or dating relationships during adolescence. It includes physical aggression, sexual violence, psychological or emotional aggression, coercive control, stalking, relational aggression, and technology-facilitated forms of abuse (Ramírez et al., 2025). This definition is intentionally broad because adolescent relationships are shaped by developmental factors, peer norms, digital communication, gender socialization, and changing expectations about intimacy. Therefore, dating violence in adolescence should not be reduced to physical or sexual acts only; it also includes patterns of control, humiliation, fear, monitoring, pressure, and normalization of harmful romantic myths (Centers for Disease Control and Prevention, 2025; Martínez Soto and Ibabe, 2022; Rothman et al., 2022; Sánchez-Jiménez et al., 2023).

This broader conceptualization has direct implications for measurement. Instruments designed for adolescent dating violence must distinguish between victimization and perpetration, capture bidirectional dynamics when relevant, differentiate behavioral domains, and remain sensitive to the developmental context of adolescents. For example, measures such as the Measure of Adolescent Relationship Harassment and Abuse (MARSHA), the Cyber Dating Violence Instrument for Teens (CyDAV-T), the Checklist of Experiences of Psychological Abuse to the Couple, and questionnaires on romantic love myths or victim-blaming attitudes show how the field has moved from a narrow behavioral focus toward multidimensional assessment of psychological, digital, attitudinal, and relational dimensions (Arrojo et al., 2024; Lara and Gómez-Urrutia, 2021; Rey Anacona et al., 2021; Rothman et al., 2022; Sánchez-Jiménez et al., 2023). Systematic evidence on technology-facilitated intimate partner violence also shows that digital behaviors are described using heterogeneous labels and measures, strengthening the case for clearer construct definitions and validation standards (Kim and Ferraresso, 2023).

Cross-cultural validation is a central but complex challenge. Many instruments are originally developed in Global North settings and later translated or validated in other countries. This process can improve comparability, but it may also reproduce conceptual asymmetries when local researchers are expected to retain the original structure and items even when they do not fully represent the cultural experience of dating, gender, control, family norms, or digital communication in their contexts (Barreiro et al., 2026). Thus, validation should not be understood only as linguistic translation; it should also examine cultural relevance, item functioning, factorial invariance, and whether adaptations are necessary to preserve construct validity. Spanish-language studies from Chile, Ecuador, and Spain demonstrate the relevance of cross-cultural validation (Lopez-Del Burgo et al., 2021; Pérez-Martínez et al., 2021), while Portuguese-language and Brazilian evidence illustrates why the discussion should extend beyond Spanish-speaking contexts (de Moura et al., 2024; Tarriño-Concejero et al., 2024). Evidence on relationship-power measurement in sub-Saharan African populations also shows that constructs and item content may not transfer uniformly across social and cultural settings (Closson et al., 2022). Moreover, psychometric work in Niger has identified gender-related differential item functioning in attitudes toward intimate partner violence, and research involving South African adolescent girls and young women shows that language-group invariance cannot be assumed even for widely used mental-health screening tools (Fonseka et al., 2023; Rodriguez et al., 2025).

Another important nuance concerns gender and sexuality. The field has historically relied on binary sex comparisons and on instruments developed mainly from cisgender and heterosexual relationship scripts. This is problematic because lesbian, gay, bisexual, transgender, queer or questioning, intersex, asexual, non-binary, and other sexual and gender identities (LGBTQIA+) adolescents may experience forms of violence that traditional measures do not adequately capture, such as threats of outing, identity invalidation, coercion linked to stigma, or control embedded in contexts of family rejection and peer marginalization. Recent reviews and empirical studies indicate that sexual and gender minority youth face elevated and specific risks of teen dating violence, while many studies still use non-validated or insufficiently inclusive measures (Kiekens et al., 2022; Ricks et al., 2023; Sulla et al., 2025).

Bibliometrics is useful in this context because it does not simply count publications; it also shows how a research field is organized, where production is concentrated, what themes dominate, and which gaps remain underdeveloped. Previous bibliometric work on intimate partner violence has shown that mapping publication patterns can identify regional inequalities, thematic saturation, and emerging areas for research and intervention (Nguyen et al., 2023).

Accordingly, this study aimed to map scientific production located at the intersection of adolescent dating violence and measurement or psychometric terminology. Specifically, the analysis examined temporal growth, concentration by source, author, institution, and country, keyword co-occurrence, conceptual structure, and underrepresented themes. The study was not designed to estimate pooled effects or to appraise the psychometric quality of individual instruments; rather, it characterizes the broader knowledge structure in which adolescent dating violence is measured and discussed.

## Methods

### Study design

A retrospective bibliometric review was conducted using a quantitative approach. Reporting was guided by the preliminary BIBLIO recommendations for bibliometric reviews and by a Preferred Reporting Items for Systematic Reviews and Meta-Analyses (PRISMA) 2020-adapted flow diagram used solely to document database retrieval, duplicate removal, and construction of the final corpus (Montazeri et al., 2023; Page et al., 2021). This design should not be interpreted as a systematic review or meta-analysis of primary psychometric studies.

### Search strategy

The search was conducted in Web of Science and Scopus on 20 March 2026. The strategy combined terms related to intimate partner violence, dating violence, adolescence, validation, invariance, psychometrics, and instruments. It was intentionally broad to map the bibliometric field surrounding adolescent dating violence measurement, rather than restricting the focus to primary instrument-development or validation studies.

Table 1 reproduces the database-specific search strategies and filters as executed and reports the corresponding retrieval counts.

**Table 1:** Database-specific search strategies and records retrieved.

Database	Search strategy and filters	Records retrieved (*n*)
Web of science	((“Partner Violence Intimate” OR “Intimate Partner Violence” OR “Violence Intimate Partner” OR “Intimate Partner Abuse” OR “Abuse Intimate Partner” OR “Partner Abuse Intimate” OR “Dating Violence” OR “Violence Dating”) AND (Adolescents OR Adolescence OR Teens OR Teen OR Teenagers OR Teenager OR Youth OR Youths OR “Adolescents Female” OR “Adolescent Female” OR “Female Adolescent” OR “Female Adolescents” OR “Adolescents Male” OR “Adolescent Male” OR “Male Adolescent” OR “Male Adolescents”) AND (Adaptation OR Validation OR Invariance OR Psychometric OR “Validation studies” OR “Validation study” OR “Validation scale” OR Scale OR Instrument))	5,143
Scopus	((“Partner Violence Intimate” OR “Intimate Partner Violence” OR “Violence Intimate Partner” OR “Intimate Partner Abuse” OR “Abuse Intimate Partner” OR “Partner Abuse Intimate” OR “Dating Violence” OR “Violence Dating”) AND (Adolescents OR Adolescence OR Teens OR Teen OR Teenagers OR Teenager OR Youth OR Youths OR “Adolescents Female” OR “Adolescent Female” OR “Female Adolescent” OR “Female Adolescents” OR “Adolescents Male” OR “Adolescent Male” OR “Male Adolescent” OR “Male Adolescents”) AND (Adaptation OR Validation OR Invariance OR psychometric OR “validation studies” OR “validation study” OR “validation scale” OR Scale OR instrument))	2,061

The search was conducted on 20 March 2026. Search strings and database-specific filters are reproduced as executed.

### Corpus construction and bibliometric procedure

The records retrieved from Web of Science (*n =* 5,143) and Scopus (*n =* 2,061) were exported and merged in *R* using the *bibliometrix* package. The combined retrieval contained 7,204 records. During database conversion and merging, the package’s duplicate-detection procedure identified and removed 1,079 duplicate records, yielding a final corpus of 6,125 unique bibliographic records. Figure 1 documents each stage of corpus construction. The unit of analysis was the bibliographic record; therefore, the findings represent publication, citation, affiliation, and thematic patterns rather than study-level estimates of instrument performance.

**Figure 1:**
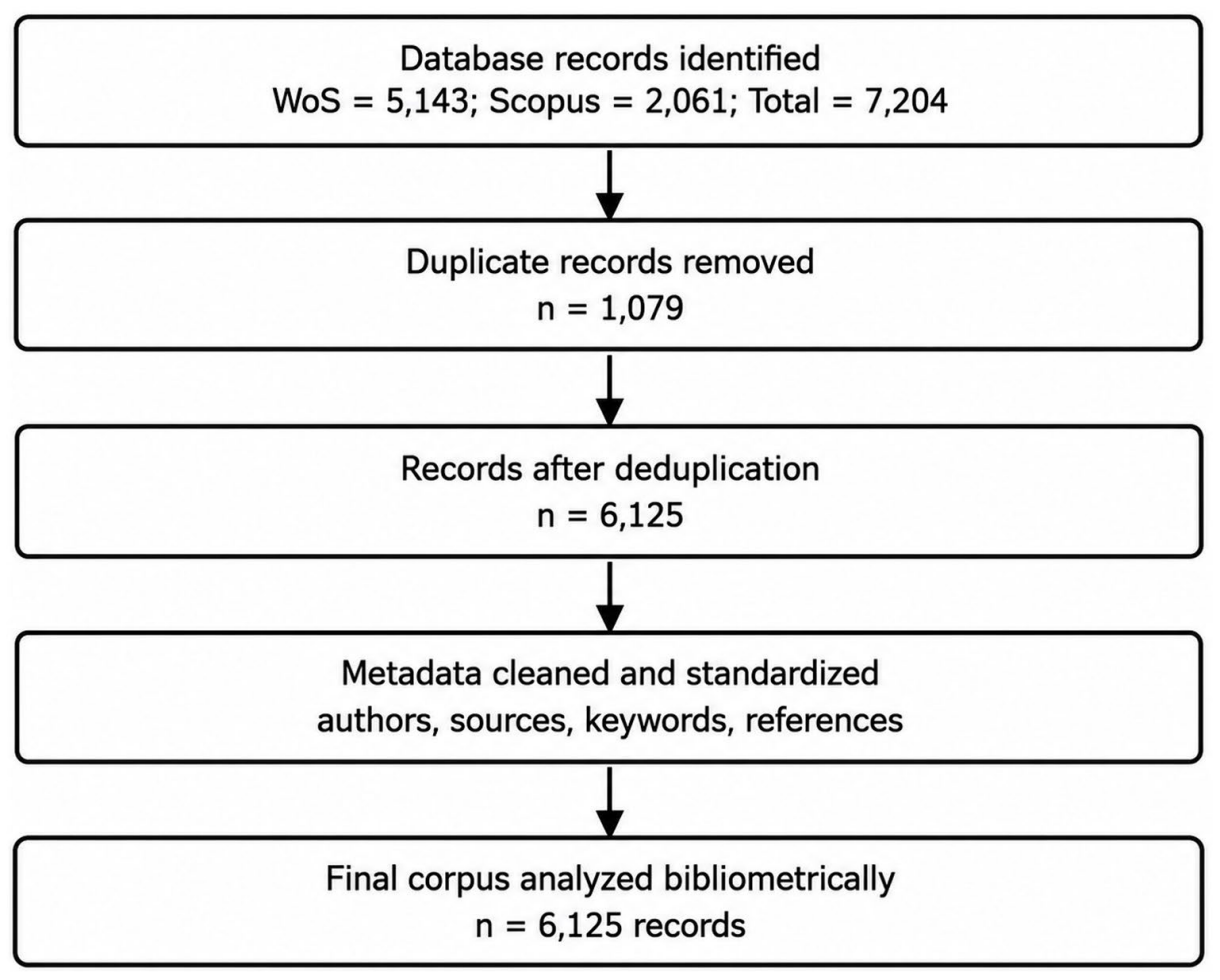
Corpus construction flow diagram for the bibliometric review. The diagram reports database retrieval, duplicate removal, metadata standardization, and the final corpus analyzed bibliometrically.

The broad strategy captured records related to instrument development, validation, psychometrics, clinical and epidemiological applications, and theoretical discussions that used measurement-related descriptors. This scope was retained to represent the broader knowledge structure surrounding the adolescent dating violence measurement. No formal risk-of-bias or methodological-quality appraisal was conducted because the review did not synthesize effect estimates or evaluate the validity of individual primary studies. Accordingly, bibliometric prominence should not be interpreted as evidence of psychometric quality, and the heterogeneity of the included records is considered explicitly in the Limitations section.

Bibliometric indicators included annual scientific production, mean citation indicators by publication year, source productivity, author-level metrics, institutional and country participation, keyword co-occurrence, and conceptual structure through multiple correspondence analysis (MCA). A logistic growth model was fitted to cumulative production: *N(t) = K/[1 + exp(−r(t − tm))]*, where N(t) is cumulative production at indexed time t, K is the projected upper asymptote, r is the growth-rate parameter, and tm is the indexed midpoint of the growth curve. Time was indexed as publication year minus 1986; thus, tm = 36.44 corresponds to approximately 2022.44. The reported R^2^ = 0.87 indicates a strong correspondence between the observed and fitted cumulative counts. K and tm were treated as descriptive model parameters rather than definitive forecasts because there were residual deviations remained and the observation for 2026 was incomplete at the time of the search.

## Results

Selected bibliographic elements differed markedly in their availability within the corpus. Cited references and science categories were available across all 6,125 records, offering the most comprehensive structural information for classifying the field. In contrast, author keywords (*n =* 852) and Keywords Plus (*n =* 812) provided complementary thematic information that requires more cautious interpretation (Figure 2).

**Figure 2:**
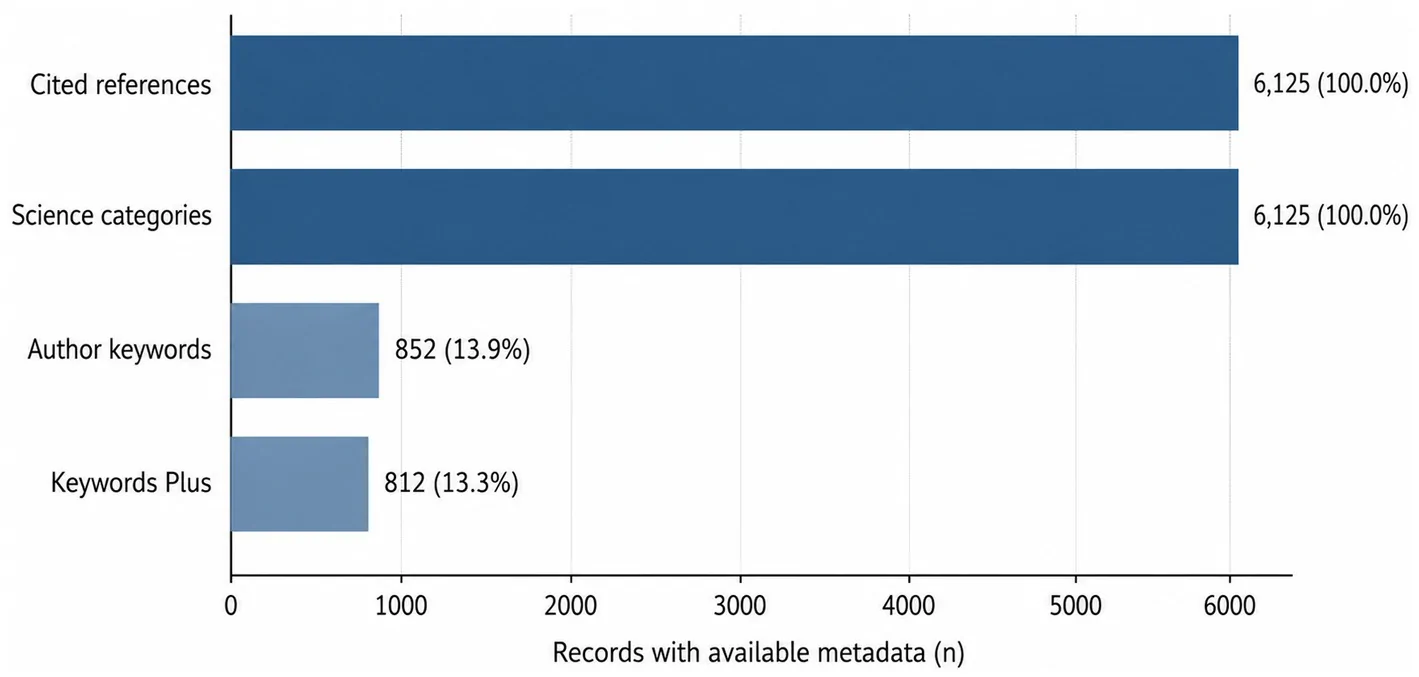
Relative availability of selected bibliographic elements in the analyzed corpus. Counts indicate the number of records with each metadata element; percentages use the final corpus (*n* = 6,125) as the denominator.

Overall, the corpus contained sufficient metadata for source-, citation-, and category-level analyses. Keyword-based results should nevertheless be interpreted as a thematic approximation because indexing practices and missing keyword fields can influence which concepts become visible in the maps.

Annual scientific production increased slowly and irregularly from 1987 through the early 2000s, indicating that adolescent dating violence measurement initially occupied a limited position within the broader interpersonal-violence literature. Production became more visible between 2006 and 2013 as research increasingly addressed adolescent relationships, psychological aggression, and the need for context-sensitive measurement tools.

The most significant increase in output occurred after 2014. In 2014, the annual output reached 263 records and continued to rise over the following decade, reaching 802 records in 2025. The Figure 802 denotes the number of records published during 2025 alone, not the cumulative production from 2014 to 2025. This trend demonstrates rapid expansion; however, the increase in publication volume should not be interpreted as evidence that the field has reached conceptual or methodological saturation.

Citation impact followed an age-dependent pattern. Earlier publication cohorts accumulated the highest mean citation values, with particularly large averages in 1996 (909.5 citations per article; 29.34 citations per year) and 1987 (630 citations per article). These values may reflect a small number of highly influential foundational papers and should not be generalized to every article published in those years.

More recent cohorts, particularly those from 2023 to 2026, showed lower mean citation counts primarily because they had less time to accrue citations and the 2026 dataset was incomplete. The coexistence of highly cited foundational publications and rapidly increasing recent output is therefore more consistent with an expanding field supported by an established intellectual base than with declining scientific relevance. The annual production and mean citation profiles are summarized in Figure 3.

**Figure 3:**
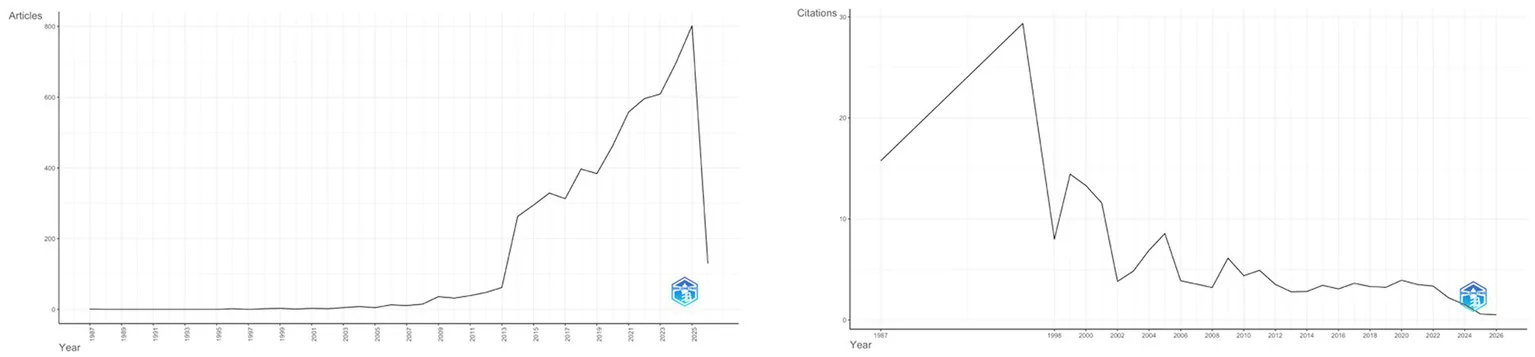
Annual scientific production and mean citation indicators by publication year in adolescent dating violence measurement research.

The fitted logistic curve provides a compact summary of cumulative production (Figure 4). The estimated upper bibliometric projection was K = 7,861.20 records. The midpoint parameter was tm = 36.44 on the time scale indexed from 1986, corresponding to approximately 2022.44. The model fit (R^2^ = 0.87) indicates that the curve provides a useful, although imperfect, summary of the observed cumulative growth pattern.

**Figure 4:**
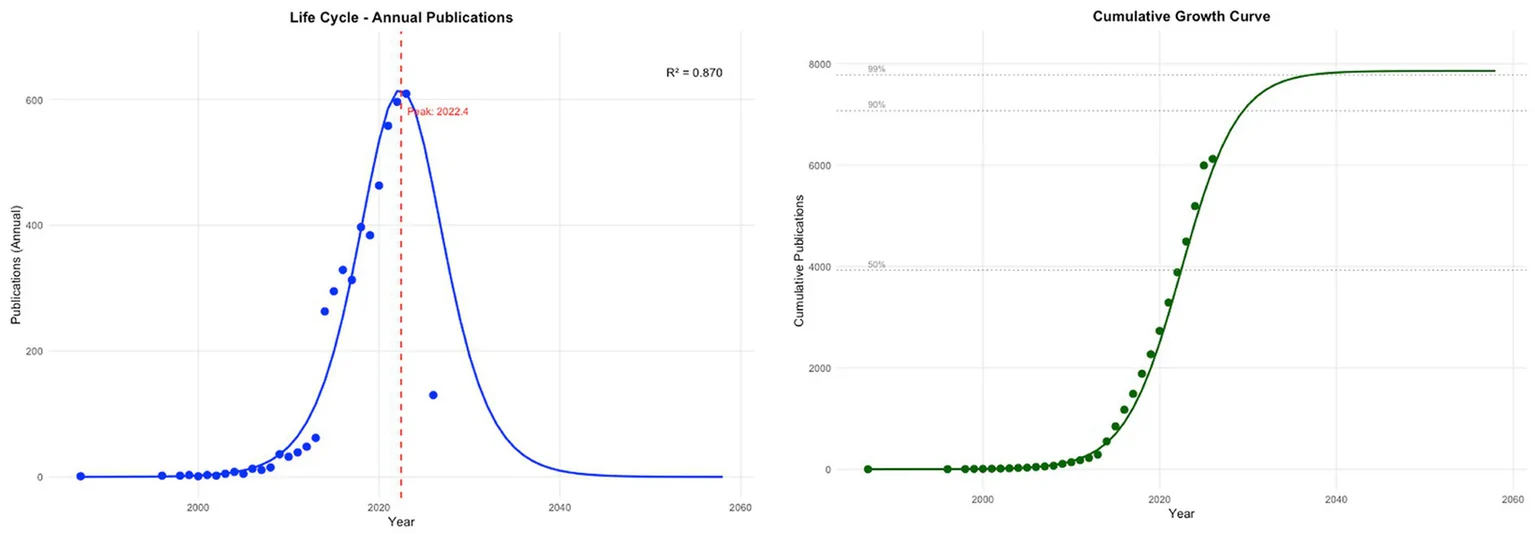
Logistic fit of cumulative bibliometric growth in adolescent dating violence measurement research.

The derivative of the fitted curve placed the most dynamic phase of growth in the early 2020s, with a model-based peak close to 615.95 records per year. This fitted peak is distinct from the observed annual maximum and does not imply that publication has ceased. Rather, it suggests that the rate of cumulative growth may be slowing after a period of pronounced acceleration.

Residual deviations were visible in several years, particularly during 2014, 2016, and the partial 2026 period. Consequently, the model supports a cautious interpretation of movement toward consolidation, rather than asserting full maturity or saturation. Additional complete publication years and updated model comparisons are needed before stronger conclusions can be drawn.

Table 2 presents the 20 most productive sources in the corpus. All percentages were recalculated using the complete corpus (*n* = 6,125) as the denominator. *Journal of Interpersonal Violence* was the leading source (*n* = 260; 4.24% of the corpus), followed by *Frontiers in Psychology* (*n* = 202; 3.30%) and *Journal of Affective Disorders* (*n* = 106; 1.73%).

**Table 2:** Twenty most productive scientific journals in the bibliometric corpus (*n* = 6,125).

No.	Source	Articles (*n*)	*%* of corpus
1	Journal of Interpersonal Violence	260	4.24
2	Frontiers in Psychology	202	3.30
3	Journal of Affective Disorders	106	1.73
4	Current Psychology	99	1.62
5	Journal of Child And Family Studies	85	1.39
6	Psychological Assessment	82	1.34
7	Frontiers in Psychiatry	81	1.32
8	Assessment	73	1.19
9	Journal of Youth and Adolescence	70	1.14
10	BMC Psychiatry	69	1.13
11	Journal of Adolescence	67	1.09
12	Child Abuse & Neglect	63	1.03
13	Violence Against Women	63	1.03
14	BMC Psychology	59	0.96
15	Child Psychiatry & Human Development	58	0.95
16	Trauma, Violence, and Abuse	58	0.95
17	European Child & Adolescent Psychiatry	53	0.87
18	Journal of Family Violence	53	0.87
19	Behavioral Sciences	51	0.83
20	Child and Adolescent Psychiatry and Mental Health	50	0.82

Together, the 20 leading journals accounted for 1,702 records, equivalent to 27.79% of the total corpus. This concentration indicates a recognizable core of publications, while also showing that the field is multidisciplinary, which includes interpersonal violence, psychology, psychiatry, adolescent development, assessment, and family violence.

Author-level indicators were interpreted as descriptive properties of the analyzed corpus rather than as evidence of the substantive authority or methodological quality of individual studies. Rankings based on h-index, g-index, total citations, and publication counts reflect visibility within this dataset. Authors listed in these rankings are not necessarily included in the reference list, which only contains works directly cited in the manuscript.

Institutional participation was concentrated in universities with established research capacity across Europe, North America, Asia, and Oceania. King’s College London (*n =* 102), Beijing Normal University (*n =* 97), and the University of Seville (*n* = 88) were among the most productive affiliations, followed by South China Normal University and the University of Melbourne (*n =* 84 each), the University of Coimbra and the University of Pennsylvania (*n* = 80 each), the University of Hong Kong and the University of Valencia (*n* = 77 each), and the University of Amsterdam and the University of California, Los Angeles (*n* = 72 each). Affiliation counts are non-mutually exclusive because a single publication can involve more than one institution.

Participation from different countries varied significantly. The United States had the highest output, while China (*n* = 1,889), Spain (*n* = 1,779), the United Kingdom (*n* = 1,112), Germany (*n* = 1,032), Australia (*n* = 931), and Canada (*n* = 921) were also major contributors. The overall counts for each country are based on author affiliations and are therefore non-mutually exclusive; records with internationally coauthorship can be counted for more than one country.

The Netherlands (*n* = 654), Italy (*n* = 614), Portugal (*n* = 435), Norway (*n* = 389), France (*n* = 345), and Sweden (*n* = 342) contributed substantially within Europe. In Latin America, Chile (*n* = 259), Brazil (*n* = 234), Mexico (*n* = 170), Colombia (*n* = 106), Ecuador (*n* = 70), and Peru (*n* = 64) were visible, although their participation remained lower than that of the leading countries.

The distribution underscores the need to strengthen research capacity and measurement visibility in underrepresented settings where dating, gender norms, digital communication, and help-seeking may have distinct cultural meanings. The global pattern of country-level production is summarized in Figure 5.

**Figure 5:**
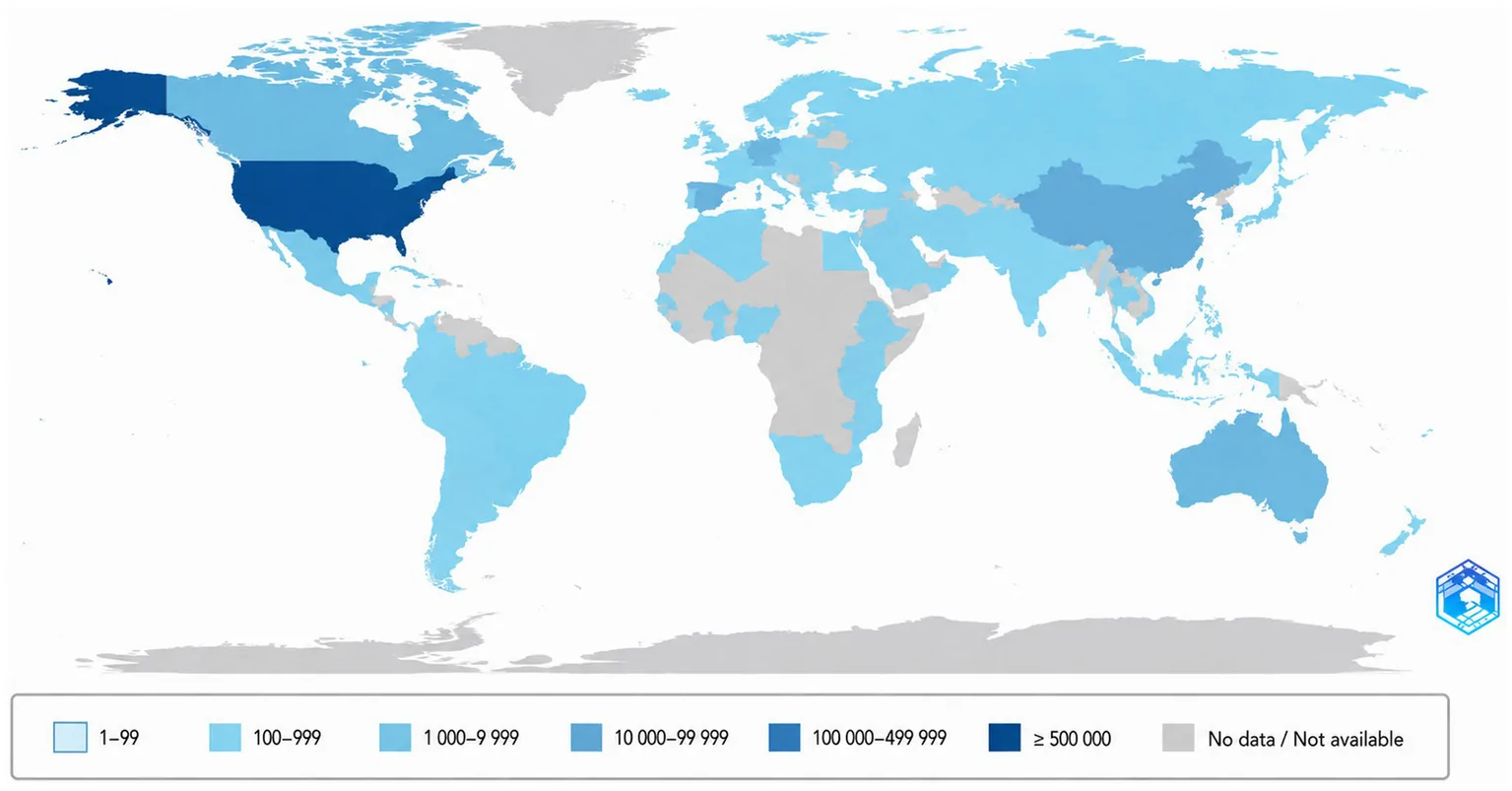
Scientific production by country in adolescent dating violence measurement research. Note. Darker shades indicate greater author-affiliation production.

Two broad thematic concentrations emerged in the keyword co-occurrence network (Figure 6). One concentration connects adolescence, validation, psychometric properties, validity, reliability, depression, anxiety, and mental health, representing a methodological–clinical axis in which measurement quality is examined alongside psychological correlates. The other concentration connects intimate partner violence, questionnaire, aggression, gender, students, and relational violence, representing a behavioral and population-oriented axis.

**Figure 6:**
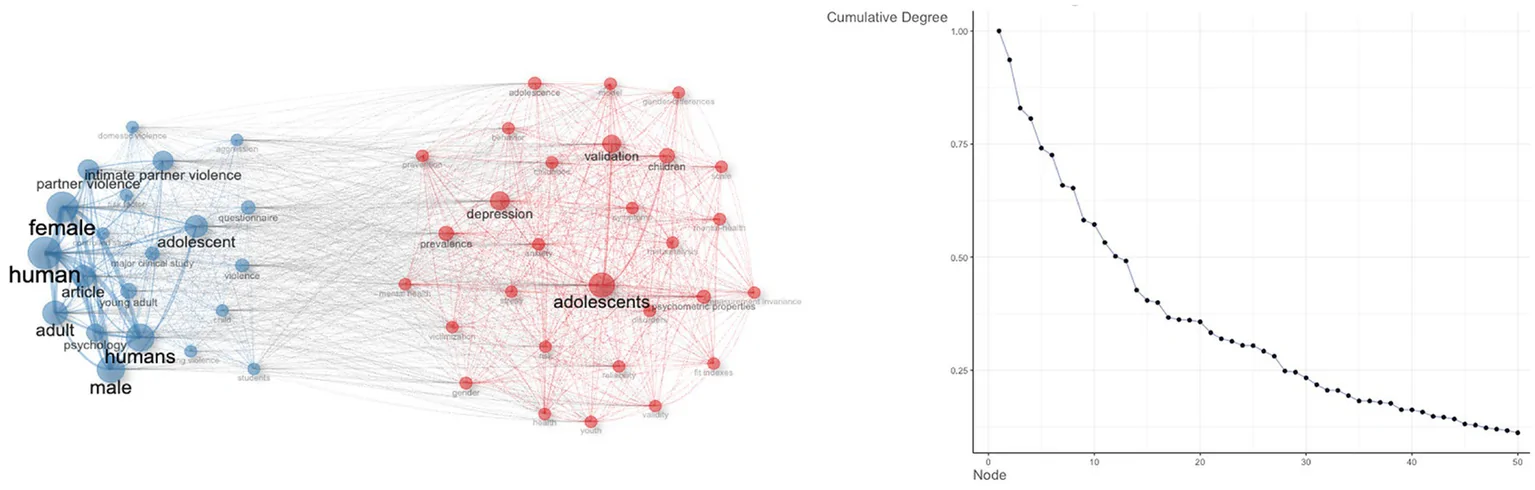
Keyword co-occurrence network and nodal-degree distribution in adolescent dating violence measurement research.

Terms such as depression, adolescents, prevalence, questionnaire, gender, anxiety, and violence appeared to bridge methodological, epidemiological, and clinical subthemes. In contrast, generic indexing labels such as human, humans, female, and male were not treated as substantive research priorities because their prominence is strongly influenced by database indexing conventions rather than by author-defined conceptual emphasis.

Conceptually, the network indicates that the literature has developed around four connected concerns: instrument validation, mental-health consequences, prevalence estimation, and gendered or relational patterns of aggression. Equally informative is what remained peripheral: terms related to sexual orientation, gender diversity, non-binary identities, and cross-cultural adaptation did not emerge as central nodes, revealing an important gap in the measurement agenda.

Modularity, network density, and average degree statistics were not available in the retained output. Cluster interpretation is therefore limited to a cautious visual and conceptual analysis of the map, and it should not be considered a definitive quantitative classification of the network.

Multiple correspondence analysis identified three broad conceptual regions (Figure 7). Unlike the co-occurrence network in Figure 6, which emphasizes pairwise term connections and node prominence, the MCA map positions terms according to similarities in their distribution across documents, highlighting broader conceptual proximity and separation. The first region grouped population and relationship descriptors, including adolescent, young adult, intimate partner violence, partner violence, psychology, and questionnaire. This region represents the general empirical context in which adolescent dating violence is investigated.

**Figure 7:**
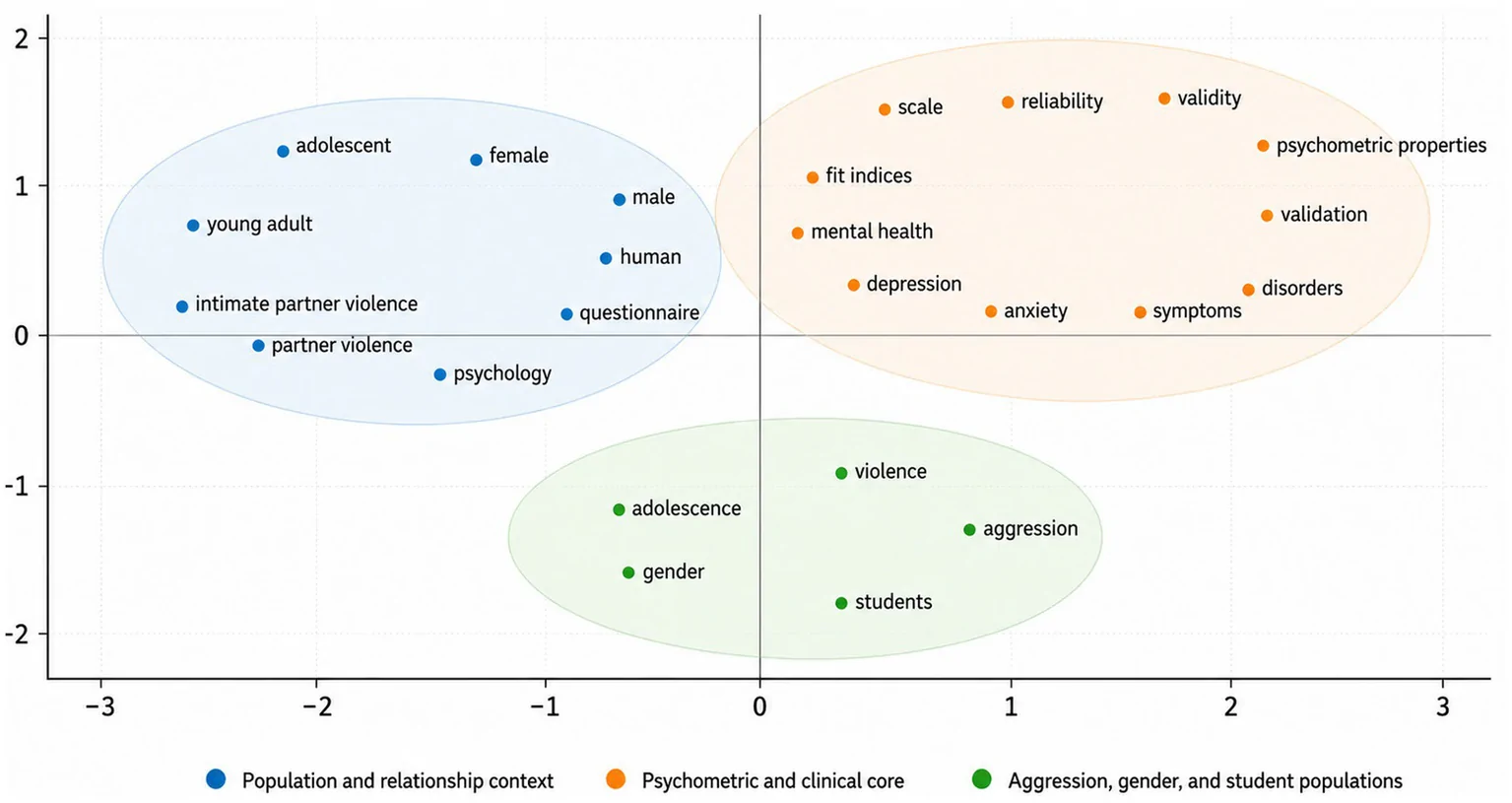
Conceptual structure of keywords using multiple correspondence analysis (MCA) in adolescent dating violence measurement research. The MCA map emphasizes broad proximity among terms across documents, whereas the co-occurrence network in Figure 6 emphasizes direct term connections and node prominence.

The second region grouped psychometric and clinical terms, including validation, psychometric properties, validity, reliability, scale, fit indices, mental health, depression, anxiety, symptoms, and disorders. This region represents the methodological core of the corpus and shows that measurement quality is frequently examined in conjunction with emotional and mental-health outcomes.

The third region included aggression, violence, adolescence, gender, and students, confirming the centrality of school and adolescent populations. However, the map continued to privilege binary gender categories and did not visibly integrate sexual orientation or gender-diverse identities. The conceptual structure thus reflects both the field’s established concerns and the limited incorporation of inclusive measurement frameworks.

## Discussion

This review identifies a field that has shifted from limited visibility to rapid expansion and increasing conceptual organization. The acceleration observed after 2014 coincides with a broader understanding of adolescent dating violence, which now encompasses not only physical and sexual acts but also psychological aggression, coercive control, relational harm, digital monitoring, and attitudes that normalize abuse. The volume of publications demonstrates sustained scientific attention, while the logistic model suggests movement toward consolidation. Nevertheless, consolidation should be understood as a provisional bibliometric pattern, not as evidence that measurement problems have been resolved.

The principal methodological contribution is a clearer definition of the corpus. The review maps literature situated at the intersection of adolescent dating violence and measurement or psychometric terminology; it does not constitute a systematic evaluation of the psychometric quality of every instrument represented. This distinction changes how the results should be interpreted. Highly productive journals, authors, institutions, or countries indicate visibility and influence within the corpus, whereas citation and network prominence do not establish validity, reliability, cultural adequacy, or clinical utility.

The geographic distribution reveals a persistent asymmetry in knowledge production. North American, European, and selected Asian institutions dominate the corpus, while Latin American and other underrepresented regions contribute a smaller share. Translation alone is insufficient to correct this imbalance. Cross-cultural validation should assess linguistic equivalence, cultural relevance, response-process validity, factorial structure, measurement invariance, and differential item functioning. It should also examine whether locally meaningful expressions of control, family influence, gender norms, peer surveillance, and digital behavior are represented adequately.

The thematic maps integrate measurement, mental health, prevalence, aggression, and gender, suggesting that instruments are commonly used to define the phenomenon and to connect it with psychological outcomes. However, the peripheral position of sexual orientation and gender-diversity terms exposes an important limitation of the field. Measures built around cisgender and heterosexual relationship scripts may fail to capture threats of outing, identity invalidation, stigma-related coercion, or barriers to disclosure and help-seeking. Therefore, inclusive measurement requires more than adding demographic response options; it requires construct development with sexual- and gender-minority adolescents and testing whether items function comparably across groups.

The findings have direct implications for research, prevention, and practice. Longitudinal evidence links childhood adversity with later adolescent intimate partner violence, while pregnancy-related stigma and partner violence are associated with anxiety and depression among adolescents in sub-Saharan African settings (Asiimwe et al., 2022; Kidman et al., 2023). In parallel, dedicated measurement of adolescent relational skills illustrates the value of assessing capacities that may inform prevention and relationship education (Fortin et al., 2021). Research should combine quantitative psychometric methods with cognitive interviewing and participatory approaches involving culturally and gender-diverse adolescents. Prevention programs should measure physical and sexual violence together with psychological control, humiliation, digital surveillance, coercion, and romantic myths. Schools, community services, and health professionals need brief, valid, and culturally responsive tools that assess both victimization and perpetration, support safe referral decisions, and avoid binary or heteronormative assumptions.

Taken together, the evidence depicts a productive but uneven field. The growth in publications and the emergence of a recognizable thematic core indicate increasing consolidation, while geographic concentration, incomplete inclusivity, and uneven cross-cultural validation show that methodological maturity is still lacking.

## Limitations

This study has several limitations. First, the intentionally broad search strategy produced a heterogeneous corpus that may include clinical, epidemiological, theoretical, and measurement-related records that are not primary psychometric validation studies. Second, the review was restricted to Web of Science and Scopus databases, so relevant literature indexed elsewhere, including regional and non-English-language sources, may be underrepresented. Third, no formal risk-of-bias or methodological-quality appraisal was performed because the unit of analysis was the bibliographic record and the objective was bibliometric mapping rather than systematic evidence synthesis. Fourth, citation and affiliation indicators were influenced by publication age, database coverage, and multi-institutional or multinational coauthorship. Fifth, keyword networks depended on metadata completeness and indexing conventions. Moreover, modularity, density, and average-degree statistics were unavailable, limiting network interpretation. Finally, 2026 data were incomplete, and the logistic parameters should be treated as descriptive projections rather than definitive forecasts of saturation.

## Conclusion

Research on adolescent dating violence measurement has expanded substantially and now constitutes a recognizable, multidisciplinary scientific domain. The bibliometric evidence shows rapid temporal growth, a concentrated core of journals and institutions, and a thematic structure linking validation, mental health, prevalence, aggression, and gender. At the same time, the field remains geographically uneven and only partially responsive to cultural and gender/sexual diversity.

Future development should prioritize culturally responsive adaptation, measurement invariance and differential item functioning across languages and social groups, and participatory instrument design with LGBTQIA*+*, non-binary, queer, and gender-questioning adolescents. Progress in these areas will improve prevalence estimation, risk detection, prevention evaluation, and professional decision-making without sacrificing local meanings or the comparability needed for cumulative scientific research.

## Data Availability

The original contributions presented in the study are included in the article/Supplementary material, further inquiries can be directed to the corresponding author.
